# Racial differences in the built environment—body mass index relationship? A geospatial analysis of adolescents in urban neighborhoods

**DOI:** 10.1186/1476-072X-11-11

**Published:** 2012-04-26

**Authors:** Dustin T Duncan, Marcia C Castro, Steven L Gortmaker, Jared Aldstadt, Steven J Melly, Gary G Bennett

**Affiliations:** 1 Department of Society, Human Development, and Health, Harvard School of Public Health, Boston, MA, USA; 2 Harvard Prevention Research Center on Nutrition and Physical Activity, Harvard School of Public Health, Boston, MA, USA; 3 Department of Global Health and Population, Harvard School of Public Health, Boston, MA, USA; 4 Department of Geography, University at Buffalo, State University of New York, Buffalo, NY, USA; 5 Department of Environmental Health, Harvard School of Public Health, Boston, MA, USA; 6 Department of Psychology and Neuroscience & Duke Global Health Institute, Duke University, Durham, NC, USA

**Keywords:** Spatial epidemiology, Neighborhood effects, Built environment, BMI, Adolescents, Race effects

## Abstract

**Background:**

Built environment features of neighborhoods may be related to obesity among adolescents and potentially related to obesity-related health disparities. The purpose of this study was to investigate spatial relationships between various built environment features and body mass index (BMI) z-score among adolescents, and to investigate if race/ethnicity modifies these relationships. A secondary objective was to evaluate the sensitivity of findings to the spatial scale of analysis (i.e. 400- and 800-meter street network buffers).

**Methods:**

Data come from the 2008 Boston Youth Survey, a school-based sample of public high school students in Boston, MA. Analyses include data collected from students who had georeferenced residential information and complete and valid data to compute BMI z-score (n = 1,034). We built a spatial database using GIS with various features related to access to walking destinations and to community design. Spatial autocorrelation in key study variables was calculated with the Global Moran’s *I* statistic. We fit conventional ordinary least squares (OLS) regression and spatial simultaneous autoregressive error models that control for the spatial autocorrelation in the data as appropriate. Models were conducted using the total sample of adolescents as well as including an interaction term for race/ethnicity, adjusting for several potential individual- and neighborhood-level confounders and clustering of students within schools.

**Results:**

We found significant positive spatial autocorrelation in the built environment features examined (Global Moran’s *I* most ≥ 0.60; all *p* = 0.001) but not in BMI z-score (Global Moran’s *I* = 0.07, *p* = 0.28). Because we found significant spatial autocorrelation in our OLS regression residuals, we fit spatial autoregressive models. Most built environment features were not associated with BMI z-score. Density of bus stops was associated with a higher BMI z-score among Whites (Coefficient: 0.029, *p* < 0.05). The interaction term for Asians in the association between retail destinations and BMI z-score was statistically significant and indicated an inverse association. Sidewalk completeness was significantly associated with a higher BMI z-score for the total sample (Coefficient: 0.010, *p* < 0.05). These significant associations were found for the 800-meter buffer.

**Conclusion:**

Some relationships between the built environment and adolescent BMI z-score were in the unexpected direction. Our findings overall suggest that the built environment does not explain a large proportion of the variation in adolescent BMI z-score or racial disparities in adolescent obesity. However, there are some differences by race/ethnicity that require further research among adolescents.

## Introduction

Globally, adolescent obesity is one of today’s most pressing public health concerns often marked by persistent racial/ethnic disparities. Racial/ethnic minority U.S. adolescents (e.g. Blacks and Hispanics) have particularly heightened rates of obesity [1,2] and nationally representative U.S. trend data indicate that racial/ethnic minority adolescents have had statistically significant increases in obesity from 1988–1994 to 1999–2000 (while non-Hispanic White adolescents have not) [1]. Though racial/ethnic disparities in obesity have been documented for well over a half-century [3], the determinants of this variation remain evasive.

Built environments of neighborhoods can have features that promote energy expenditure (e.g. by facilitating or impeding physical activity) as well as energy intake (e.g. through its influence on food availability). Thus, built environmental features of neighborhoods may play a role in the increases of obesity among adolescents, and in known obesity-related racial/ethnic health disparities. Indeed, adolescents are more independent than younger children, potentially making them more susceptible to environmental conditions, and several reviews have shown that racial/ethnic minority populations have increased exposure to built environment features that can contribute to obesity; disparities in the built environment might be an explanation for obesity-related racial/ethnic health disparities among adolescents and other populations [4,5]. A number of studies show that neighborhoods that have access to walking destinations (such as recreational facilities and parks) and that have ‘walkable’ community designs (such as sidewalks, increased number of intersections and a high density of residences) are associated with favorable obesity-related outcomes (especially increased physical activity) among adolescents [6,7]. Although there is a burgeoning literature in this area, it is significant to note that much less research has examined features of the built environment as related to adolescent BMI specifically; most of the existing studies in this area have focused on adolescent physical activity [6,7]. The limited available research that has examined relationships between features of the built environment and BMI among adolescents has resulted in inconsistent findings and several studies showed no significant effects [6,7].

Most studies that examine influences of the built environment on adolescent BMI have not conducted analyses considering the possibility of racial/ethnic variation in the effects. It is possible that aggregate models can ‘mask’ important relationships for certain population subgroups and can also increase the likelihood of non-significant findings (if the associations between the built environment and obesity risk vary by race/ethnicity in terms of magnitude, statistical significance and/or direction of effect) [8]. Furthermore, most of the existing studies in this area have a limited number of racial/ethnic minority populations in the sample—restricting the generalizability of their findings and also the power for any subgroup analysis by race/ethnicity. Recent reviews on the built environment and adolescent BMI have called for additional studies with ‘diverse’ populations as related to race/ethnicity [6] and for additional studies that consider moderators (such as race/ethnicity) in the relationship between the built environment and adolescent BMI [7]. A small but growing literature is explicitly exploring whether the built environment might be a factor in disparities in obesity. A recent review—in which most of the studies published focused on adults—showed that the built environment is associated with obesity risk among racial/ethnic minority populations [4] though a previous review found that the built environment had less consistent associations among racial/ethnic minority populations [9]. Critical unanswered questions remain regarding relationships between policy-relevant features of the built environment and BMI among adolescents, especially regarding how built environment neighborhood features might be implicated in obesity-related racial/ethnic health disparities [6,7].

Methodological difficulties when analyzing relationships between the built environment and BMI remain problematic, including regarding potential racial/ethnic differences in these effects. First, neighborhoods have been defined differently across studies, and as indicated by the modifiable areal unit problem, this likely leads to different results in the literature [10,11]. Second, assuming independence of individuals from different neighborhoods, most studies evaluating relationships between built environmental features and obesity risk among adolescents neglect to examine and account for the spatial connections between neighborhoods, i.e., how neighboring areas are related to each other, although the possible presence of spatial effects (e.g. spatial dependence) can influence the results in meaningful ways. Indeed, similar to the majority of the neighborhood effects literature, several studies examining the influence of the built environment on adolescent BMI followed a traditional multi-level modeling approach, which treats neighborhoods as disconnected areas. However, neighborhoods are not spatially isolated and previous research has shown that multilevel models do not necessarily account for spatial autocorrelation [12,13]. Emerging research indicates that spatial clustering of obesity might exist [14-19], raising questions about factors leading to these potential clusters and also indicating that spatial regression methods may be appropriate [20-24]. Third, a sample of adolescents from different racial/ethnic groups is needed to examine racial/ethnic differences, and a sample with a sizeable number of adolescents from racial/ethnic minority groups is needed if researchers are particularly interested in those population subgroups.

The goal of this study was to investigate spatial relationships between various built environment features and BMI z-score among a sample of adolescents across the city of Boston who predominantly come from racial/ethnic minority groups, and to investigate whether race/ethnicity modifies the studied relationships using geospatial analysis techniques such as spatial autoregressive models, if necessary. A secondary objective was to evaluate the sensitivity of results to defining neighborhoods at different spatial scales in order to better understand the spatial scale aspect of the modifiable areal unit problem (MUAP)—which is arguably the most troublesome aspect of MAUP. As such, this research seeks to address the limitations of past studies.

## Methods

### Study design and sample

**Figure 1:**
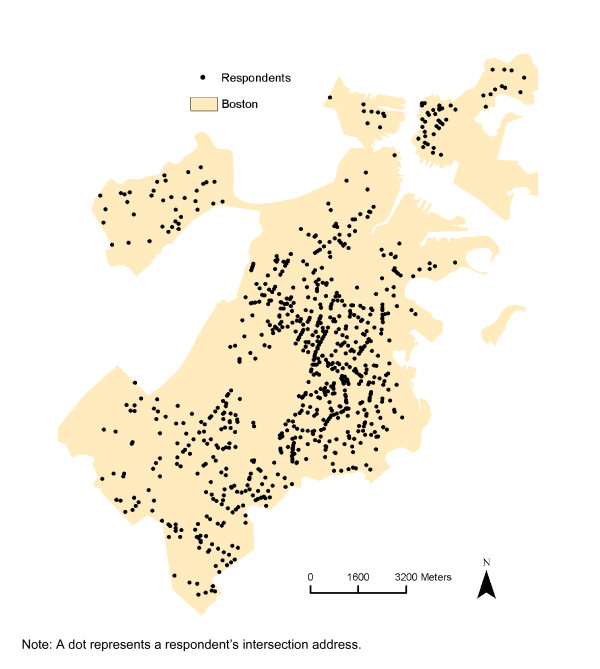
Spatial Distribution of the Sample, 2008 Boston Youth Survey Geospatial Dataset (n = 1,034).

Data for this study came from the 2008 Boston Youth Survey (BYS), a survey of 9^th^-12^th^ grade students in the Boston Public Schools system [25]. Approximately 74% of Boston Public School students in the 2007–2008 academic year were eligible for free or reduced-price meals [26], similar to the percentage of those schools included in the BYS survey [27]. Religious schools, private schools and other schools not within the purview of the Boston Public School system are not included. Schools that served adults, students transitioning back to school after incarceration, suspended students and students with severe disabilities were ineligible. All 32 eligible public high schools in Boston were invited to take part in the study in 2008; 22 participated. The primary reason for school non-participation was scheduling difficulties (e.g. conflicts with mandatory standardized testing). There were no statistically significant differences in key school characteristics (e.g. racial/ethnic composition of students, proportion of students receiving free or reduced price lunches, drop-out rates, standardized test scores or student mobility rate) across participating and non-participating eligible schools. To generate our sample, we assembled a list of unique classrooms within each participating school, stratified by grade and randomly selected classrooms for survey administration. Every student within the selected classrooms was invited to participate. Selection of classrooms continued until approximately 100–125 students had been sampled per school. The survey was administered to students by trained staff in the spring of 2008. Students completed the questionnaire during the allotted 50-minute class periods. Passive consent was sought from parents and students were read a statement regarding assent prior to survey administration. Of the 2,725 students enrolled in the classrooms selected for participation, 1,878 completed a survey (response rate = 68.9%). Students who did not complete a survey either: (a) chose not to participate (3.6%), (b) were not permitted by a parent to take the survey (1%), or (c) were absent from school on the day of survey administration (26.6%). Of the students selected for participation and present on the day of the survey (n = 2001), 93.9% completed surveys. We obtained complete address information to the nearest intersection from 68.8% of the Boston students who took the survey (n = 1,292). Two-hundred fifty-eight of these students were missing complete and valid data for computing BMI z-score, and thus were not included in the sample, resulting in a final sample of 1,034 students. There was not a statistically significant difference in biologically plausible BMI z-scores (BMI calculations are discussed below) between students who provided complete intersection residential addresses and those who did not. Figure 1 shows a map of the study area and the spatial distribution of these respondents.

### Address geocoding

To geocode the neighborhoods in which the BYS students live, but not compromise confidentiality, students were asked to provide the name of the street on which they live and the nearest cross-street in addition to other geographic information (e.g. zip code) [25]. All addresses were preprocessed before geocoding by systematically and extensively cleaning them to improve their quality. We reviewed the data for misspelled street names and checked them to ensure that the address existed using Google Maps, remedying incorrect addresses (e.g. incorrect street names and streets that did not intersect) or multiple addresses listed, when necessary. In addition, we standardized spelling to the United States Postal Service format (e.g. we changed ‘Street’ to ‘St’, ‘Avenue’ to ‘Ave’, and ‘Circle’ to ‘Cir’). After excluding participants not located in Boston (n = 17), addresses were geocoded to the street intersection and assigned longitude and latitude coordinates, using the Environmental Systems Research Institute (ESRI) Street Map USA address locator, which uses a U.S. Census Bureau TIGER 2000 streets dataset enhanced by ESRI and Tele Atlas for the reference layer, published in 2006, with ArcGIS version 9.3 (ESRI, Redlands, CA). The resulting points were imported into Google Maps, where each location was checked for accuracy and refined. Addresses that ArcMap failed to recognize, but which were real intersections, were manually placed on the map in Google Maps. The verified points were imported back into ArcMap for processing.

### Built environment features

We built a geospatial database that includes various built environment features with ArcGIS 9.3 software using the Massachusetts state plane projection North American Datum (NAD) 1983. This study included the following variables related to access to walking destinations: recreational open space per square kilometer, parks per square kilometer, bus stops per square kilometer, subway stops per square kilometer, total retail walking destinations (e.g. clothing stores, pharmacy/drug stores, bookstores) per square kilometer, total service walking destinations (e.g. post offices, banks, credit unions) per square kilometer and total cultural/educational walking destinations (e.g. movie theaters, schools, libraries) per square kilometer. We limited the retail, service and cultural/educational walking destinations to locations with fewer than 250 employees to filter out large businesses (e.g. Costco’s, Home Depot’s) as businesses with more than 250 employees may reduce the walkability of neighborhoods (e.g. by having large parking lots) [28] and for comparability with previous published research evaluating built environment correlates of obesity risk among adolescents [29]. Recreational open space and public transit data as of 2007 come from the Office of Geographic Information (MassGIS), Commonwealth of Massachusetts, Information Technology Division, the state agency responsible for the collection, storage and dissemination of publicly available geographic data for Massachusetts [30]. Parks data come from ESRI Data and Maps 2006; and retail, service and cultural/educational walking destinations data come from ESRI Business Analyst InfoUSA Business Locations 2006. ESRI Data and Maps information, from ESRI, has geospatial datasets representing various built environment features. InfoUSA [31] is a proprietary information service; the company provides listings of private and public businesses (verified yearly by telephone), with 6-digit NAICS codes as well as numbers of employees. Locations of these businesses had been geocoded and were available as a geospatial dataset through the ESRI Business Analyst Extension. We also included the following variables related to community design: median pedestrian route directness (median of the ratio of distance between one point and another via the street network and straight-line distance between the two points; values closer to 1.00 represent a more direct route or a more connected network), intersection density (the number of street intersections per square kilometer; intersections are defined as street network nodes with 3 or more associated street segments excluding highways), sidewalk completeness (excluding sidewalks in parks, informal paths and cut-throughs and excluding roads with medians; sidewalk completeness was calculated using the following equation: [left sidewalk length + right sidewalk length]/total road length times 100 divided 2; thus, a 0 is no sidewalk and a 100 indicates presence of sidewalk on both sides), average sidewalk width in meters (same exclusions), average speed limit (miles per hour), highway density (percentage of area that is highway traveled right of way; highways are defined as primary roads with limited access or interstate highways) and residential density (US census block group occupied housing units per square kilometer were weighted proportionally for the adolescents’ defined neighborhood). Median pedestrian route directness data come from ESRI Business Analyst InfoUSA Business Locations 2006; intersection density and average speed limit data come from ESRI Data and Maps Street Map 2006; sidewalk availability and highway density data come from the MassGIS 2007; and residential density data as previously described come from the 2000 US Census. We used ‘ego-centric’ neighborhood definitions in this study, not administrative boundaries (e.g. zip codes or census tracts) because increasingly buffer-based neighborhood definitions (i.e. a buffer around a study participants residential address) are used in neighborhood effects on health research and buffer-based neighborhood definitions are likely to be more relevant to young people’s social realities and health [32]. We specifically defined the adolescent’s neighborhood as 400- and 800-meter street network buffers for two primary reasons. First, these distances are considered a proximal neighborhood environment for adolescents [33], including an appropriate independent walking distance for them [34]. Second, street network buffers, in comparison to circular buffers, are more relevant to human geography (i.e. human travel patterns) because they take into account the street geography and impermeable barriers. Indeed, research shows that they are more predictive of physical activity than circular buffers [35]. The street network buffers were created from StreetMap streets excluding highways and ramps using the ArcGIS Network Analyst Extension. The street network buffers consisted of 50-meter buffers around street center lines that extend along the network 400- and 800-meters from the geocoded residential addresses.

### Body mass index

BMI was calculated using students’ answers to items on height and weight, i.e., weight in kilograms divided by height in squared meters. Biologically implausible heights, weights and BMI values were dropped prior to any analysis (n = 40). Specifically, we dropped outlier variables for height-for-age, weight-for-age, weight-for-height and BMI-for-age based on Centers for Disease Control and Prevention (CDC) growth charts from the year 2000 [36], which were created using SAS version 9.2 and CDC SAS growth chart programs for computing anthropometric values [37]. Based on the 2000 CDC growth charts, we converted BMI to z-scores accounting for age and gender norms. BMI z-score was used in this study because it is a more appropriate measure of adiposity than BMI for adolescents [38]. A BMI *z*-score is the number of standard deviation units that an individual’s BMI is from a population mean value.

### Individual and neighborhood-level covariates

Individual-level covariates include: race/ethnicity (non-Hispanic White, non-Hispanic Black, Hispanic, Asian and Other), gender (male, female), age (years), nativity (U.S. born, foreign-born) and other youth in household (yes, no). Neighborhood-level covariates include: percent of non-Hispanic Black residents, percent of Hispanic residents, percent of households below poverty level and percent foreign born. Neighborhood-level measures were based on 2000 US Census Data and were interpolated proportionally based on the census block groups for the adolescents’ defined neighborhood (values across block groups were weighted proportionately by each block group’s area within the defined buffer).

### Geospatial analysis

#### Exploratory spatial data analysis

After having performed descriptive statistics for the individual and neighborhood characteristics, we conducted exploratory spatial data analysis, i.e. geovisualization and cluster detection. Using ArcGIS, geovisualization was conducted to map features of the built environment and BMI z-score. This facilitated an initial inspection of potential spatial patterns. We constructed maps to show the spatial distribution of the built environment features and BMI z-score among the sample (map colors were based on Color Brewer 2.0) [39]. We present maps of all built environmental features for the 800-meter buffers to demonstrate the different levels of spatial autocorrelation and variation in the patterns in features of the built environment. The Jenks natural breaks classification method, which determines the best grouping of values in the data, was used when mapping the built environment features. This method reduces the variance within classes, while maximizing the variance between classes [40]. For BMI z-score, we created a standard deviation map, showing how much variation there is from the mean BMI z-score. We assessed the presence of overall spatial dependence in built environment features and BMI z-score with the Global Moran’s *I* statistic, which is the most commonly used test statistic for spatial autocorrelation [22,24]. For the Global Moran’s *I* calculations and all subsequent spatial regression models, we specified a k nearest neighbor (KNN) spatial weights matrix. A value is one if the neighboring spatial units are ‘neighbors’ and zero if ‘not neighbors’. KNN was chosen as the structure for spatial relationships because: (a) we wanted all respondents to have an equal number of neighbors; (b) this specification represents the influence of one’s most immediate neighbors; and (c) this specification results in everyone having neighbors [41]. We specifically used a k nearest neighbor spatial weights matrix specification of four, because it has previously been suggested that a spatial weights matrix specification between four and six neighbors is optimal and because it is accepted that applying an under-specified (fewer neighbors) rather than an over-specified (extra neighbors) weights matrix is better (e.g. for increased power) [42,43]. The associated pseudo p-value of the Global Moran’s *I* was calculated through a Monte Carlo simulation consisting of 999 random replications. Moran’s *I* values range between −1 to 1. A Moran’s *I* value near 0 indicates a lack of spatial pattern (values observed at one location do not depend on values observed at neighboring locations). This is the null hypothesis of complete spatial randomness. Positive coefficients reflect neighboring areas with similarly large or small values (similarity or positive spatial autocorrelation). Negative coefficients reflect neighboring areas with large inverse values (dissimilarity or negative spatial autocorrelation). One potential reason for spatial clustering, or spatial autocorrelation, is shared predictor variables that cluster in space. Investigating the presence of spatial autocorrelation in BMI z-score, both descriptively through mapping and statistically through cluster detection, provides preliminary evidence for spatial regression modeling.

### A-spatial and spatial regression analysis

For our continuous outcome data, BMI z-score, we fit ordinary least squares (OLS) regression models. If the OLS regression residuals had significant spatial autocorrelation, we applied a well-known spatial econometric approach for spatial regression modeling by fitting spatial simultaneous autoregressive error models (hereafter referred as the spatial error model), estimated via maximum likelihood [20-24]. Recognizing that there are different techniques to estimate spatial linear regression models [44], we conducted a preliminary sensitivity analysis via the generalized method of moments estimation of the spatial error model [44,45], however, the estimates and p-values replicated were near identical as the initial standard maximum likelihood estimation approach and histograms of the residuals for the spatial error model estimated via maximum likelihood showed normally distributed residuals, indicating that the maximum likelihood approach was correct (we therefore present findings from using the maximum likelihood approach). The spatial error model accounts for spatial autocorrelation by including an autoregressive term for the error structure based on a specified spatial weights matrix [20-24]. Because less is empirically known about the use of asymmetric spatial weights matrices (e.g. point A is B's nearest neighbor but point B might not be point A's nearest neighbor) when estimating spatial autoregressive models [44], we converted the asymmetric KNN spatial weights to make it symmetric (our preliminary modeling though showed that the two spatial weights matrices produced similar results). The average number of neighbors for the asymmetric spatial weights matrix was 5.31 (range 4 to 11; 98.1% had 4 to 8 neighbors). The distances for the specified spatial weights matrix ranged from 0 to 1526 meters (mean range for the KNN 4 spatial weights matrix was 191.10 meters; mean range for the KNN 4 symmetric spatial weights matrix was 210.00 meters). Using the KNN 4 symmetric spatial weights matrix, the Global Moran’s *I* statistic and the Lagrange Multiplier test for the spatial error model were used to evaluate the fitted OLS regression residuals for evidence of spatial autocorrelation [20,23,46,47]. The Global Moran's *I* statistic is applied to the error terms of the OLS model to assess spatial autocorrelation. The Lagrange Multiplier test for spatial error dependence can be used when the Moran's *I* is statistic significant. If the Lagrange Multiplier test for the spatial error model is significant that should be the proper specification for the data. If spatial models were necessary, the fit of the OLS and spatial error models were compared using the Akaike Information Criterion (AIC). The AIC examines overall model fit and model complexity; lower AIC values indicate a better fit. Finally, if spatial models were fit, we conducted a spatial Hausman test comparing the magnitude of the OLS and spatial error model parameter estimates based on the null hypothesis of correct specification [20,48]. Each model estimating relationships between features of the built environment and BMI z-score was conducted using the total sample (Model 1) as well as including an interaction term between the built environment feature and race/ethnicity (Model 2) in order to assess potential effect modification in the studied relationship. We included the interaction term in the models (as opposed to conducting stratified analysis) not only to formally evaluate effect modification but also because in models including an interaction term the spatial matrix is of the entire sample (when conducting stratified analysis the spatial weights matrix is only for that strata, which might not be appropriate). Separate models were run for each built environment feature to examine their unique contribution on BMI z-score and due to expected multicollinearity between the features of the built environment examined. To evaluate the sensitivity of neighborhood effects, these models were fit for our two neighborhood definitions: 400- and 800-meter street network buffers. All models controlled for the previously described individual- and neighborhood-level confounding factors selected based on past theoretical and empirical research. School was included as a fixed effect to control for clustering of students within schools. All data analyses were conducted using the R statistical program version 2.12 with Bivand’s *spdep* package [44].

## Results

### Sample characteristics

Sample characteristics are provided in Table 1 for the total sample and disaggregated by race/ethnicity. Respondents were predominantly non-Hispanic Black (42%) and Hispanic (33%) and the mean age was 16.3 years. Over half were female and just over one-quarter of the sample was born outside of the US. The majority had at least one other youth living in their home. Although there was racial/ethnic variation, the mean BMI z-score was 0.51. Based on the CDC classifications, 33.17% of the adolescents were overweight or obese (BMI percentile 85 or greater). Table 2 provides descriptive statistics for the various featfres in the built environment. Because respondents’ lived in neighborhoods across the city, it is not surprising that there is variation in the built environment features. It is important to highlight though that there are small standard deviations for several built environment features—indicating that there is not much dispersion from the mean for several of these features.

**Table 1:** Sample Characteristics, 2008 Boston Youth Survey Geospatial Dataset by Race/Ethnicity

	**Total (n = 1,034)**	**White (n = 107)**	**Black (n = 428)**	**Hispanic (n = 330)**	**Asian (n = 78)**	Other* (n = 72)
BMI z-score (mean, SD)	0.51 (1.08)	0.50 (1.08)	0.55 (1.13)	0.66 (0.99)	−0.19 (1.10)	0.37 (1.03)
Age in years (mean, SD)	16.32 (1.26)	16.20 (1.19)	16.39 (1.27)	16.24 (1.24)	16.63 (1.29)	16.18 (1.31)
Gender (%)						
Male	44.29	54.21	43.46	45.76	42.31	27.78
Female	55.71	45.79	56.54	54.24	57.69	72.22
Nativity Status (%)						
US Born	73.73	88.79	73.82	70.34	59.74	88.73
Foreign Born	26.27	11.21	26.18	29.66	40.26	11.27
Other youth in household (%)						
Yes	85.48	84.31	86.45	85.35	85.53	81.43
No	14.52	15.69	13.55	14.65	14.47	18.57

*Includes non-Hispanic youth who were bi- or multi-racial, American Indian or Alaska Native, Native Hawaiian or Other Pacific Islander, or youth who did not fit into any of the specified race categories.

Note: Percentages may not total 100 due to rounding.

**Table 2:** Built Environment Features: Descriptive Statistics and Spatial Autocorrelation

	**400-meter Network Buffer Neighborhood**			**800-meter Network Buffer Neighborhood**		
	**Mean (SD)**	**Range**	**Moran’s *I*^a^**	**Mean (SD)**	**Range**	**Moran’s *I*^a^**
**Access to Walking Destinations**						
Recreational open space (density)	4.71 (4.27)	0 - 21.11	0.79	3.71 (2.66)	0 - 16.42	0.88
Parks (density)	2.85 (2.83)	0 - 17.24	0.70	2.10 (1.32)	0 - 8.21	0.79
Bus stops (density)	25.81 (13.07)	0 - 63.36	0.66	25.37 (8.02)	0 - 47.56	0.83
Subway stops (density)	0.57 (1.64)	0 - 14.84	0.59	0.60 (1.30)	0 - 11.43	0.88
Retail destinations (density)	18.97 (21.27)	0 - 230.70	0.74	18.24 (15.02)	0 - 159.10	0.83
Service destinations (density)	1.64 (3.68)	0 - 42.32	0.63	2.01 (4.06)	0 - 71.45	0.67
Cultural/educational destinations (density)	14.48 (12.90)	0 - 154.30	0.77	15.33 (11.67)	0 - 128.60	0.90
**Community Design**						
Median pedestrian route directness	1.14 (0.16)	1.00 - 2.73	0.15	1.18 (0.15)	1.00 - 3.12	0.38
Intersection density	113.21 (33.96)	23.86 - 305.90	0.86	105.43 (26.60)	48.41 - 262.80	0.94
Sidewalk completeness	85.31 (12.08)	10.47 - 100.00	0.68	84.11 (10.23)	17.62 - 99.47	0.83
Average sidewalk width (meters)	1.84 (0.33)	0.20 - 2.70	0.77	1.81 (0.28)	0.30 - 2.43	0.88
Average speed limit (mph)	28.00 (1.71)	24.78 - 39.40	0.76	27.82 (1.19)	25.00 - 35.34	0.89
Highway density	0.55 (2.42)	0 - 26.48	0.69	0.65 (2.08)	0 - 16.59	0.84
Residential density	375.45 (210.87)	52.76 - 1488.00	0.93	353.15 (172.45)	59.48 - 1194.00	0.96

^a^ The pseudo p value for the Global Moran's *I* are all 0.001.

Note: All density measures are expressed as per square kilometer.

### Spatial distribution and spatial autocorrelation in the built environment and BMI z-score

**Figure 2:**
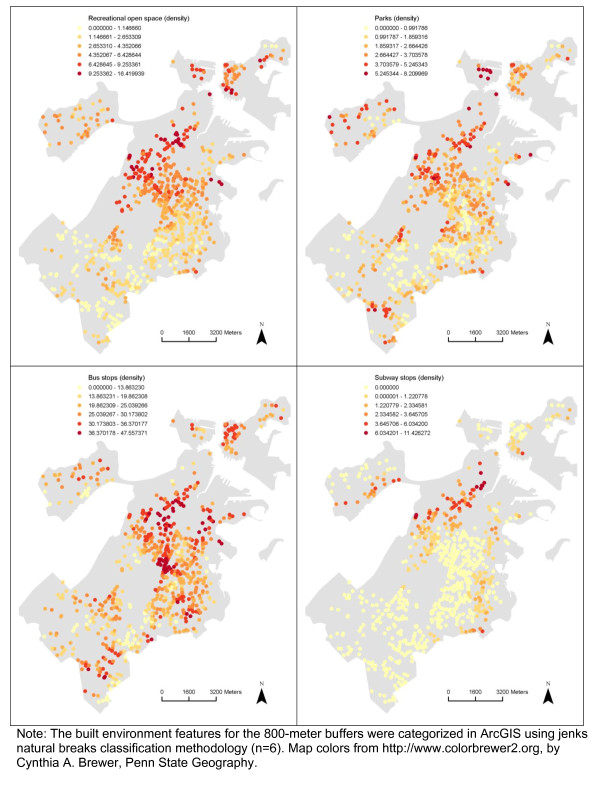
Spatial Distribution of Recreational open space, Parks, Bus stops and Subways stops among the Sample, 2008 Boston Youth Survey Geospatial Dataset (n = 1,034).

**Figure 3:**
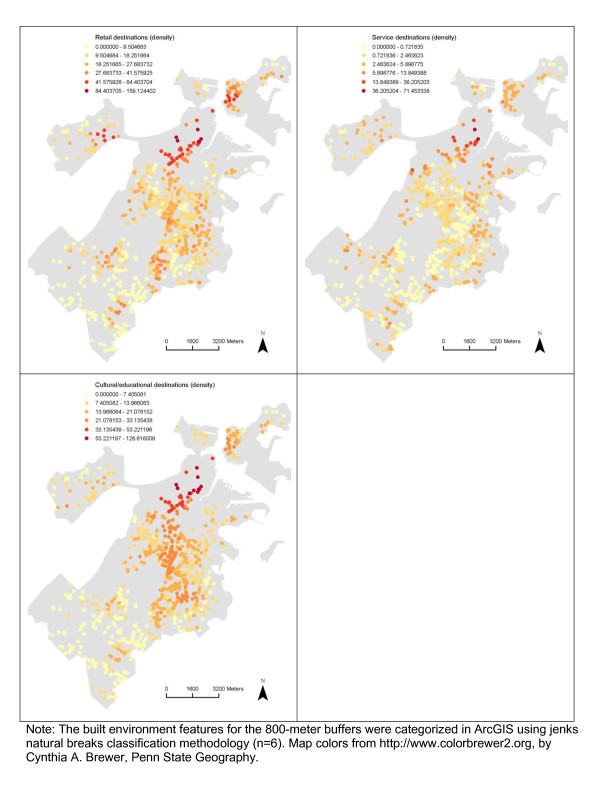
Spatial Distribution of Retail destinations, Service destinations and Cultural/education destinations among the Sample, 2008 Boston Youth Survey Geospatial Dataset (n = 1,034).

**Figure 4:**
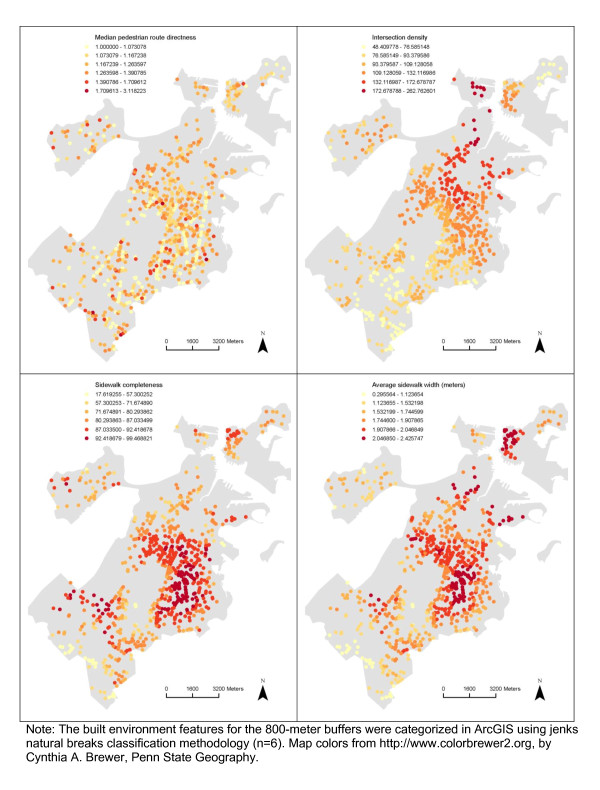
Spatial Distribution of Median pedestrian route directness, Intersection density, Sidewalk completeness and Average sidewalk width among the Sample, 2008 Boston Youth Survey Geospatial Dataset (n = 1,034).

**Figure 5:**
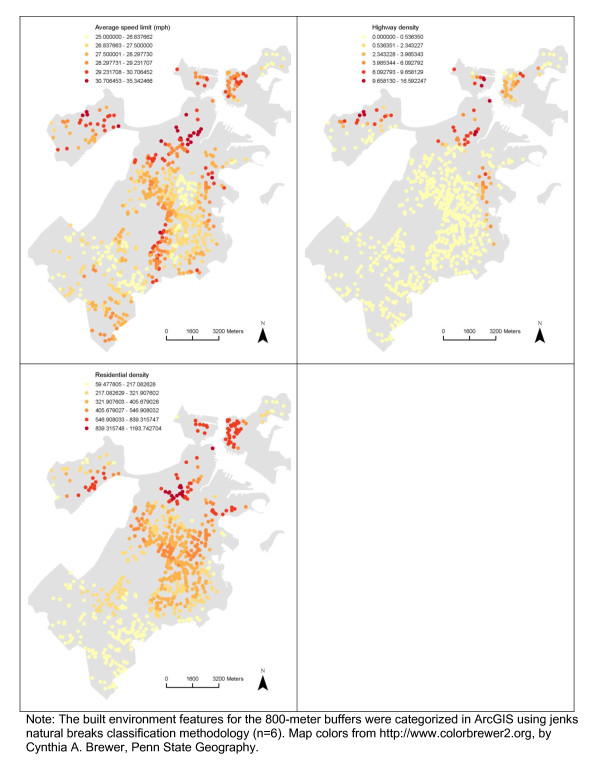
Spatial Distribution of Average speed limit, Highway density and Residential density among the Sample, 2008 Boston Youth Survey Geospatial Dataset (n = 1,034).

**Figure 6:**
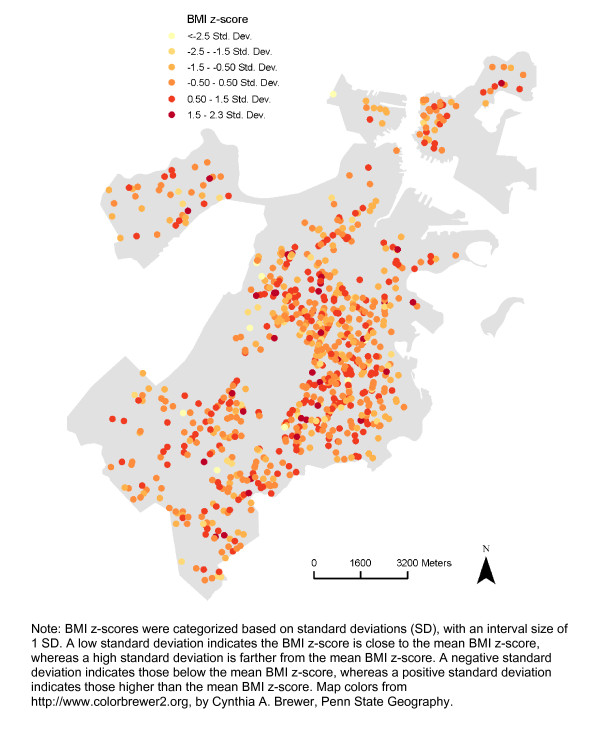
Spatial Distribution of BMI z-scores among the Sample, 2008 Boston Youth Survey Geospatial Dataset (n = 1,034).

There appeared to be spatial patterns in features of the built environment for each neighborhood definition (Figures 2, 3, 4, 5). This was confirmed statistically via the Global Moran’s *I* statistic. The Global Moran’s *I* value for most built environment features were ≥ 0.60 (indicating strong positive spatial autocorrelation) and were all statistically significant (*p* = 0.001) (Table 2). The geography of adolescent BMI z-score in Boston is shown in Figure 6. While there was tremendous local variation in adolescent BMI z-scores across Boston neighborhoods, there did not appear to be any overall patterns spatially in BMI z-score based on geovisualization. The Global Moran’s *I* for BMI z-score was 0.07 (*p* = 0.277).

### Spatial relationship between features of the built environment and BMI z-score

The Global Moran’s *I* evaluating spatial autocorrelation in the OLS regression residuals for the association between features of the built environment and BMI z-score indicated that there was significant positive spatial autocorrelation (Global Moran’s *I*: all approximately 0.05, all *p* < 0.01). The Lagrange Multiplier test for spatial error model indicated that there was significant spatial autocorrelation across models (most *p* < 0.01, all *p* < 0.03). For example, in the OLS multivariate association between recreational open space and BMI z-score for the total sample based on the 800-meter network buffer, the Global Moran’s *I* was 0.052 (*p* < 0.002) and the Lagrange multiplier test p-value for the spatial error model was statistically significant (*p* = 0.013). Therefore, to take spatial autocorrelation into account, we utilized the spatial error model.

The AIC values for the spatial error models were slightly higher compared to OLS models and the additional spatial autoregressive coefficient in the spatial error model for spatial autocorrelation was not significant across models. For example, in the multivariate association between recreational open space and BMI z-score for the total sample based on the 800-meter network buffer, the OLS model AIC was 2887.3 while the spatial error model AIC was 2889.0. In this model, the spatial coefficient was 0.028, with a p-value of 0.575. Results from the spatial Hausman test, however, were non-significant—indicating that the spatial error model is an appropriate specification for these data. Due to the significant presence of spatial autocorrelation in the OLS regression residuals and the findings based on the spatial Hausman test, the spatial error model was considered to be more appropriate.

Table 3 shows the multivariate results from the spatial models estimating the association between built environment features and adolescent BMI z-score for the 800-meter network buffer. We present the results based on the 800-meter network buffer because all multivariate results for the 400-meter network buffer were not statistically significant. There was not a statistically significant association between density of recreational open space and BMI z-score. The interaction term for recreational open space for Asians was marginally significant and indicated an inverse association. There was a marginally significant interaction term in the relationship between park density and BMI for Blacks (*p* = 0.069), suggesting a positive association. Density of bus stops was significantly associated with a higher BMI z-score among Whites (Coefficient: 0.029, *p* < 0.05); the interaction term was marginally significant for Blacks, Hispanics and ‘Others’—with results showing different directions across racial/ethnic groups. In addition, service destinations and cultural/educational destinations were not associated with BMI z-score overall, however, the interaction term for Asians regarding service destinations was marginally significant (indicating an inverse association). The interaction term for Asians in the association between retail destinations and BMI z-score was statistically significant (Interaction Coefficient: -0.014, *p* <0.05) and also indicated an inverse association. Sidewalk completeness was significantly associated with a higher BMI z-score among the total sample of adolescents (Coefficient: 0.010, *p* < 0.05). Average sidewalk width might be associated with BMI z-score, though the direction of effect may vary by race/ethnicity. BMI z-score was not associated with subway stop density, median pedestrian route directness, intersection density, highway density or residential density.

**Table 3:** **Spatial Error Model Estimation of the Relationship Between Built Environment Features and BMI z-score, 800-meter Network Buffer**^a^

**Access to Walking Destinations**			**Community Design Attributes**		
	**Coefficient**	**SE**		**Coefficient**	**SE**
**A. Recreational open space (density)**			**A. Median pedestrian route directness**		
Model 1			Model 1		
A: Total Sample	−0.027	0.017	A: Total Sample	−0.384	0.243
Model 2			Model 2		
A: White	−0.005	0.039	A: White	−1.124	0.757
A X Black	0.011	0.046	A X Black	0.938	0.860
A X Hispanic	−0.036	0.044	A X Hispanic	0.765	0.837
A X Asian	−0.098~	0.059	A X Asian	0.798	1.242
A X Other	−0.018	0.063	A X Other	0.644	1.259
**B. Parks (density)**			**B. Intersection density**		
Model 1			Model 1		
B: Total Sample	0.034	0.030	B: Total Sample	0.000	0.002
Model 2			Model 2		
B: White	−0.035	0.072	B: White	0.001	0.003
B X Black	0.155~	0.085	B X Black	0.003	0.004
B X Hispanic	−0.009	0.085	B X Hispanic	−0.003	0.004
B X Asian	0.085	0.104	B X Asian	−0.005	0.005
B X Other	0.163	0.124	B X Other	0.001	0.006
**C. Bus stops (density)**			**C. Sidewalk completeness**		
Model 1			Model 1		
C: Total Sample	0.004	0.005	C: Total Sample	0.010*	0.004
Model 2			Model 2		
C: White	0.029*	0.016	C: White	0.015	0.010
C X Black	−0.027~	0.016	C X Black	−0.004	0.011
C X Hispanic	−0.029~	0.016	C X Hispanic	−0.013	0.012
C X Asian	−0.015	0.020	C X Asian	0.005	0.016
C X Other	−0.038~	0.023	C X Other	0.007	0.019
**D. Subway stops (density)**			**D. Average sidewalk width**		
Model 1			Model 1		
D: Total Sample	−0.039	0.034	D: Total Sample	0.267~	0.145
Model 2			Model 2		
D: White	−0.020	0.085	D: White	0.567~	0.321
D X Black	−0.007	0.103	D X Black	−0.156	0.378
D X Hispanic	0.019	0.101	D X Hispanic	−0.582	0.389
D X Asian	−0.063	0.098	D X Asian	−0.561	0.480
D X Other	−0.052	0.116	D X Other	−0.022	0.645
**E. Retail destinations (density)**			**E. Average speed limit**		
Model 1			Model 1		
E: Total Sample	−0.001	0.002	E: Total Sample	−0.029	0.035
Model 2			Model 2		
E: White	0.007	0.006	E: White	0.127	0.106
E X Black	−0.011	0.008	E X Black	−0.208~	0.119
E X Hispanic	−0.003	0.007	E X Hispanic	−0.112	0.117
E X Asian	−0.014*	0.007	E X Asian	−0.203~	0.123
E X Other	−0.013	0.013	E X Other	−0.167	0.161
**F. Service destinations (density)**			**F. Highway density**		
Model 1			Model 1		
F: Total Sample	−0.007	0.009	F: Total Sample	−0.017	0.020
Model 2			Model 2		
F: White	0.012	0.013	F: White	0.017	0.042
F X Black	−0.055	0.034	F X Black	−0.059	0.070
F X Hispanic	−0.015	0.031	F X Hispanic	−0.039	0.054
F X Asian	−0.036~	0.020	F X Asian	−0.040	0.052
F X Other	−0.088	0.060	F X Other	−0.060	0.105
**G. Cultural/educational destinations (density)**			**G. Residential density**		
Model 1			Model 1		
G: Total Sample	0.001	0.003	G: Total Sample	0.000	0.000
Model 2			Model 2		
G: White	0.006	0.007	G: White	0.000	0.000
G X Black	−0.002	0.009	G X Black	−0.000	0.001
G X Hispanic	−0.002	0.010	G X Hispanic	−0.000	0.001
G X Asian	−0.013	0.008	G X Asian	−0.001	0.001
G X Other	−0.008	0.017	G X Other	−0.000	0.001

*SE* =Standard Error

~ *p* < 0.10; * *p* < 0.05 (bold); ** *p* < 0.01 (bold).

^a^ Model 1 estimates the association between the built environment and BMI z-score among the total sample; Model 2 estimates the studied association and includes an interaction for race/ethnicity. For each model, we evaluate the estimated effect of each built environment feature separately. All models are adjusted for individual-level race/ethnicity, individual-level gender, individual-level age, individual-level nativity, individual-level family structure (other youth in household), neighborhood-level percent of Black residents, neighborhood-level percent of Hispanic residents, neighborhood-level percent of households below poverty and neighborhood-level percent foreign born for the 800-street network buffer. Regression estimates are also controlled for school using indicator variables.

Note: All density measures are expressed as per square kilometer.

## Discussion and conclusion

The adolescent obesity pandemic remains a public health concern. While adolescents from all U.S. racial/ethnic groups have been affected, obesity is nearly normative among certain racial/ethnic minority adolescents. In this study, we utilized a geospatial perspective to evaluate relationships between features of the built environment and BMI z-score among a citywide sample of Boston adolescents by explicitly including the spatial context within which neighborhoods are embedded. We also evaluated if race/ethnicity modified these relationships and considered the effect of different neighborhood scales in the relationships under investigation. We found that there was significant positive spatial autocorrelation in the built environment features examined but not in BMI z-score. Because we found significant spatial autocorrelation in our OLS regression residuals, we fit spatial regression models (though these spatial models did not significantly improve the model fit). In this study, some relationships between the built environment and adolescent BMI z-score were in the unexpected direction. Our findings overall suggest that built environment variables did not explain much of the variability in standardized BMI among adolescents or racial disparities in adolescent obesity but there are some differences in these relationships by race/ethnicity. However, we do not want to over interpret the interaction terms for the racial/ethnic groups due to relatively small samples sizes among certain groups. Although most built environment features were not associated with BMI z-score, density of bus stops was associated with a higher BMI z-score among Whites. The interaction term for Asians in the association between retail destinations and BMI z-score was statistically significant and indicated an inverse association. Sidewalk completeness was significantly associated with a higher BMI z-score among the total sample. These significant associations were found for the 800-meter buffer. To the best of our knowledge, this is the first study to examine relationships between various built environment features and adolescent BMI z-score to have explicitly considered geospatial issues such as spatial autocorrelation and one of few studies to consider racial/ethnic differences in effects and neighborhood effects at multiple spatial scales.

Few studies have examined spatial clustering of the built environment in residential neighborhoods, and the existing studies all show positive spatial autocorrelation in various features of the built environment [49-53]. Several studies have shown spatial clustering of obesity [14-19], but others have shown that obesity does not cluster [54,55]. The difference between our findings and those of some other studies that suggest obesity clusters could be explained by the fact that most other studies examined obesity clustering among adults (in this study we examined BMI z-score clustering among adolescents). Also, most of the studies evaluating spatial patterns in obesity used large administrative areas, whereas we evaluated spatial patterns with individual-level geocoded residential addresses. Furthermore, we measured global spatial autocorrelation (since we were interested in modeling associations and not identifying specific potential locations of spatial clustering). Global spatial autocorrelation might not exist if there is highly localized clustering [56]. To illustrate, a high prevalence of obesity might exist is a small region of the overall geographic area under investigation, but these spatial patterns in BMI might not be detected with global spatial autocorrelation tests. Several of these aforementioned studies used global spatial statistics in evaluating spatial autocorrelation in obesity [16,17,54,55]; some did not find any clustering with the global cluster detection method [54,55]. However, when assessing local clustering in obesity significant spatial patterns in obesity were found [14-16,18,19]. It therefore remains a possibility that our data could have local patterns in BMI. However, such an assessment is beyond the scope of the present study.

Only a handful of existing studies have examined built environment correlates of BMI among adolescents and only recently have studies used GIS to quantify the built environment. Several findings from our study are consistent with several of these existing studies. For example, adolescent BMI was not associated with recreational facilities and parks in several past studies [57-63]. However, a few other studies indicate an inverse association of recreational facilities and parks with adiposity among adolescents [55,64,65]. In a study among adults in Los Angeles County and southern Louisiana that examined racial/ethnic differences in associations between the built environment and BMI, access to parks was significantly associated with decreased BMI among Whites, but increased BMI among Blacks (though the latter association was not statistically significant) [66]. In a study of Massachusetts children and adolescents, public transit (particularly subway density) was inversely associated with BMI [60]. Additionally, previous research has found that intersection density were not associated with adolescent BMI [58,62,63,67]. Contrary to our findings, past research found no association between sidewalks and adolescent BMI [60]. In previous research, residential density was not associated with BMI or obesity among adolescents [55,57,62,63,67,68], although a recent study found that higher population density was associated with lower BMI in adolescents across spatial contexts [67]. We are not aware of any research examining the effect of the average speed limit on adolescent BMI, but some relevant research has been conducted. Indeed, car traffic was longitudinally associated with increased BMI among adolescents in one study [69], but was not associated with adolescent BMI in another longitudinal analysis [58]. In a cross-sectional analysis traffic danger (i.e. the ratio of roads with higher speed limits and traffic volumes to all other roads) was not associated with adolescent BMI [59] nor was traffic density cross-sectionally associated with BMI among a sample of adolescents from Kiel, Germany [57]. Finally, no association was found between road density and adolescent BMI [67].

There are several important caveats to note regarding studies examining the relationship between built environment features and adolescent adiposity. Most of the existing studies did not examine or account for potential spatial autocorrelation in regression models and most studies also did not examine race/ethnicity or neighborhood definition as potential effect modifiers. It is plausible that some of our results may differ from the existing studies due to the aforementioned reasons but also the samples in most of the existing research were not predominantly Black and/or Hispanic, as was our sample. Moreover, it is difficult to compare our findings regarding potential effect modification by neighborhood definition because several studies used buffers of different scales and zones than ours. In the present study, the significant findings pertain to the 800-meter street network buffer as compared to the 400-meter street network buffer. Of the studies with similar spatial scales, some found significant associations at larger spatial scale (about 800 meters) [55,67], though others did not at this spatial scale [61,62]. In contrast to our findings, some studies found significant associations at a smaller spatial scale (about 400 meters) [60,64,69], though these studies included a wider age range including children and adolescents, or younger adolescents only. Of note, there might be a threshold effect in the relationship between the built environment and adolescent BMI, as a very large spatial scale (1 mile or greater) for built environment features was not associated with adolescent BMI across studies [58,63] .

Because several results were unexpected, in addition to consulting the literature, we conducted post-hoc analyses and also used Google – which can be a useful resource for evaluating the neighborhood environment [70-73] – to determine plausible explanations for our findings. The finding that density of parks might be associated with higher levels of BMI among Black adolescents was unexpected; parks are generally thought to be places where people can partake in various physical activities [74]. Previous studies have found socioeconomic and racial/ethnic inequalities in the quality of parks [75-77]. There is racial residential segregation in Boston [78,79], and it is possible that the parks in Black neighborhoods have worse conditions (e.g. less safe and more trash) than the parks in certain other neighborhoods. In post-hoc analyses, Blacks in our sample were among the least likely to have recently used parks and other open spaces (which was queried in the survey) and this is consistent with previous research [80-83]. As shown in other previous cross-sectional studies [60,84] and a recent natural experiment [85], public transit was inversely associated with BMI, perhaps a result of walking to and from public transit (increasing daily physical activity level via utilitarian exercise) [86,87] and because public transportation might also be an overall indicator of urban neighborhood walkability with increased destinations [60]. Although it is known that racial/ethnic minorities (e.g. Blacks and Hispanics) walk more than their White counterparts to public transit [86], it is unclear why we found a significant positive association between bus stop density and BMI among White adolescents. It is possible that Whites might be less likely to use public transit (e.g. buses), but it is still unclear why this association exists. We speculate that the effect found might be a marker for another environmental feature (e.g. crime which can happen at bus stops) [88,89] and our 2006 survey showed that youth feel particularly unsafe on public transit (including buses) [25]. Additionally, bus stops might be associated with increased traffic noise [90], which may be associated with BMI. However, it is still unclear why this was only significant for Whites. Also, we did not expect to find that sidewalk completeness would be associated with higher BMI z-scores. In post-hoc analyses, we re-estimated the associations between sidewalk completeness and adolescent BMI z-score, controlling for all the individual- and neighborhood-level variables previously noted and also now controlling for average speed limit and highway density (as they might be associated with both the exposure and outcome). The results, however, did not appreciably change. We suspect that there may still be residual confounding and note that our sidewalk effect in the unexpected direction should be considered in light that the data was unable to account for the quality of sidewalks. Using Google Street View, we examined the streetscape and sidewalk conditions for the locations of a small number of adolescents in our sample with varying degree of sidewalk completeness. The conditions of neighborhoods with a low percentage of sidewalk completeness and neighborhoods with a high percentage of sidewalk completeness were surprisingly generally comparable in our preliminary Google investigation.

It is important to note that several of the non-significant effects might be due to some ubiquity of the neighborhood exposures; lack of a good degree in variation for adolescents on some features might limit the ability to find significant effects, especially in urban environments such as Boston. Even if there was greater variation for most of the sample, non-significant effects could still be found due to the potential counterbalancing effects of the built environment on adolescent BMI, as neighborhoods can have features that dually promote increased physical activity as well as increased food intake, which can be implicated in the spatial behavior and therefore health of neighborhood residents. For example, it is plausible that adolescents in neighborhoods with increased intersections walk (potentially resulting in energy expenditure) to stores to consume energy dense foods (potentially resulting in energy intake), which can result in energy balance—not or minimally affecting their adiposity. Some emerging work has suggested that there may be a ‘corner store phenomenon’, whereby the purchases made in corner stores can contribute significantly to energy intake among urban children [91]; these children might be making purchases at corner stores walking in their neighborhoods as well as to and/or from school. In regards to our counterintuitive bus stop density-BMI z-score finding among Whites, bus stops can be near food stores. Therefore, bus stop density could serve as a proxy for the food environment, increased energy intake and higher adiposity. Lastly, it is necessary for us to comment on our findings regarding model specification. Spatial autocorrelation was found in our standard OLS regression models, but the spatial error models did not result in improved goodness-of-fit. We speculate that this to be the case because while there was significant spatial autocorrelation in the OLS regression residuals, the magnitude of the effect was not large.

Although this study had several strengths (e.g. understanding the modifiable areal unit problem, examining race/ethnicity as a potential effect modifier, using socially meaningful neighborhoods, using GIS, providing a detailed description of our address geocoding methods and using various spatial statistical methods), it also had some limitations. The use cross-sectional analysis is a limitation, as it precludes any causal inference. Despite this limitation, our built environment exposures precede the outcome, BMI z-score, and several longitudinal studies [55,64,69,92] and natural experiments [85,93] show that features of the built environment can impact changes in obesity risk among adolescents and adults. Though the gold standard is to collect objectively measured height and weight data, this was not practical nor a central focus of the parent study. In the BYS, we had self-reported height and weight data for BMI, which can be associated with inaccurate reporting. However, past research has found that adolescents can provide valid reports of height and weight. For example, among adolescents in a previous study, the correlation between measured and self-reported height was 0.94 and the correlation between measured and self-reported weight was 0.95 (all statistically significant) [94]. Accuracy of exposure and outcome data location is important in spatial analysis, including for visualization, cluster detection and spatial regression modeling. Problems with GIS datasets can exist [95,96]; the few studies that have examined the validity of GIS built environment databases indicate that there can be errors in them [60,97,98]. Any pattern of error in a GIS dataset is likely biased towards the null when examining associations between the built environment and health [98]; this also serves as an additional potential explanation of our overall null findings. We also note that we had access to various GIS datasets to create a wide range of built environment features—including access to local GIS datasets (used in the study) which are likely to be more valid than national GIS datasets. It is still possible that there is some location misclassification. Also, because we obtained intersection addresses there may be some location misclassification and some spatial overlap in the sample. Location misclassification can produce biased estimates and reduce the statistical power to detect true associations. However, the effect of using intersections on location misclassification is likely to be small, since all study participants live within the city of Boston, which generally has a dense street network with small block sizes (so, the distance between the exact address and the intersection is likely to be quite small; thus, built environment variables at intersections are expected to be similar to variables at mid-block). Overlap in the neighborhoods of these students (meaning same intersections) can introduce nuisance spatial autocorrelation (i.e. artificially increase clustering) among independent variables. However, address information was obtained at the intersection level for several specific reasons (e.g. for confidentiality reasons and to increase the response rate); geocoding intersection addresses has been suggested as a suitable alternative to geocoding a participant’s specific residential address [99]. Importantly, the BYS geospatial sample is smaller than the adolescents who completed the survey; this is a limitation, however, there were no differences by BMI z-score in our data with regards to who provided geocodeable information and who did not, which is consistent with another recent study among adolescents [67], so ‘geographic bias’ is not an issue [100]. In this study, we control for a number of potential confounding variables at both the individual- and neighborhood-level. However, due to expected high rates of non-response, we did not seek to ascertain information on parent’s socioeconomic position (e.g. income and employment status) as well as adolescents’ residential stability, so we were unable to control for these variables in adjusted regression models. We speculate that including family-level socioeconomic variables in the analyses (if we could) would further attenuate the results, although probably not by a large degree, because the sample is predominantly low-income urban adolescents, so there likely is not as much variation in family-level socioeconomic conditions as there likely could be with samples including individuals from a broader socio-economic spectrum. Residual confounding (due to the effect of not including family-level socioeconomic variables) likely is not as much of a concern in this study as it might be in other research. Confounding by neighborhood self-selection is also a possibility (e.g. it is possible that physically active people may move to neighborhoods where there are parks and recreational facilities) [5,101-103]. However, residential selection bias might not be much of a concern in this study because it is less plausible that adolescents chose the neighborhoods that they live in (we recognize though that their parents’ still did and this could influence the adolescent’s behavior/health). Furthermore, it is also important to note that adolescents might not use resources in neighborhoods that can be related to energy imbalance. Though we found some significant racial/ethnic differences, statistical power for the racial/ethnic interaction analysis is a limitation of this study due to small samples sizes for some groups. Also, results were not corrected for multiple comparisons. These findings might only be generalizable to low-income adolescents in comparable urban locations at comparable spatial scales as the study was conducted in the city of Boston among a sample of low-income high school students using specific spatial scales.

While the findings for this study overall suggest that the built environment is not a major contributor to adolescent obesity or racial disparities in adolescent obesity, we believe that further research is needed including in other geographic locations. Given the lack of evidence found in this study that the built environment contributes substantively to adolescent obesity and obesity disparities, future research should not only consider the built environment analyzing separate measures but consider analyzing composite measures of the built environment as well as consider other explanations for adolescent obesity disparities—including perhaps an examination of the social environment (e.g. crime, neighborhood disorder). Qualitative studies can be helpful in understanding these relationships, which may vary by race/ethnicity. In addition to examining *access* to the built environment, future studies should ascertain and examine the *quality* of built environment features. There are a variety of existing tools (which can be integrated into a geospatial dataset) that facilitate examining conditions and cleanliness of the built environment [95] such as the conditions of park and open space features (e.g. the Environmental Assessment of Public Recreation Spaces [EAPRS]) [104]. An additional suggestion for future research is to query *use* of built environment neighborhood resources. While additional cross-sectional studies will also be important to gaining a greater understanding of how policy-relevant built environment features might influence obesity risk among adolescents, to address the complexity of causation in this area of research, prospective cohort designs in addition to experimental research designs can also be conducted and would provide clear temporal ordering. These future studies examining associations between the built environment and BMI should pay special attention to differences by race/ethnicity and neighborhood definition. Understanding the role that race/ethnicity may play in relationships between various built environment features and adolescent adiposity (which require large sample sizes for the racial/ethnic groups to have power to detect effects) may contribute to remedying racial/ethnic health disparities. Because neighborhood definition (e.g. spatial scale) probably always matters in neighborhood effects research, if possible, it will be useful for future studies to conduct analysis with multiple neighborhood definitions in the same study to further understand the modifiable areal unit problem. Critical thought should be given when defining neighborhoods. The appropriateness of the neighborhood definition(s) likely varies based on the process studied. Use of spatial regression methods in research examining the built environment and BMI can be important if there is spatial autocorrelation in regression residuals. Although the spatial error model is usually an appropriate spatial model, given that the source of spatial autocorrelation is often unknown, future research can consider the use of other spatial regression methods. There is also a need for additional research to further understand the role of residential self-selection as it might bias associations between the built environment and adolescent BMI; a number of approaches can be used to do this [105-107]. Among the published studies investigating the effects of the built environment on adolescent BMI, to our knowledge, only one has performed a validation of their GIS datasets [60]. The paucity of information on the validity of GIS built environment data (which may vary by location and provider) warrants additional research. Future studies using georeferenced data can also consider employing spatial sampling techniques to equalize the number of respondents at different levels of exposure and to also ensure socio-demographic heterogeneity [108-110]; use of spatial sampling can improve the quality of analysis on the built environment and obesity risk. These additional studies will expand the main findings from our study that suggest the built environment does not explain a large proportion of the variation in adolescent BMI z-score or racial disparities in adolescent obesity, although some differences by race/ethnicity existed among adolescents.
